# Effects of Microvesicles Derived from NK Cells Stimulated with IL-1β on the Phenotype and Functional Activity of Endothelial Cells

**DOI:** 10.3390/ijms222413663

**Published:** 2021-12-20

**Authors:** Kseniia Markova, Valentina Mikhailova, Yulia Milyutina, Andrey Korenevsky, Anastasia Sirotskaya, Veronika Rodygina, Elizaveta Tyshchuk, Polina Grebenkina, Andrey Simbirtsev, Sergey Selkov, Dmitry Sokolov

**Affiliations:** 1 Department of Immunology and Intercellular Interactions, Federal State Budgetary Scientific Institution, Research Institute of Obstetrics, Gynecology, and Reproductology Named after D.O. Ott, 199034 St. Petersburg, Russia; mva_spb@mail.ru (V.M.); milyutina1010@mail.ru (Y.M.); a.korenevsky@yandex.ru (A.K.); nastya.marinena@ya.ru (A.S.); corbiepost@yandex.ru (V.R.); tyshhuk.elizaveta@gmail.com (E.T.); grebenkinap@gmail.com (P.G.); selkovsa@mail.ru (S.S.); falcojugger@yandex.ru (D.S.); 2 State Research Institute of Highly Pure Biopreparations, 197110 St. Petersburg, Russia; a.s.simbirtsev@hpb.spb.ru

**Keywords:** microvesicles, microparticles, interleukin-1, inflammation, NK cells, endothelium

## Abstract

Microvesicles (MVs) are plasma extracellular vesicles ranging from 100 (150) to 1000 nm in diameter. These are generally produced by different cells through their vital activity and are a source of various protein and non-protein molecules. It is assumed that MVs can mediate intercellular communication and modulate cell functions. The interaction between natural killer cells (NK cells) and endothelial cells underlies multiple pathological conditions. The ability of MVs derived from NK cells to influence the functional state of endothelial cells in inflammatory conditions has yet to be studied well. In this regard, we aimed to study the effects of MVs derived from NK cells of the NK-92 cell line stimulated with IL-1β on the phenotype, caspase activity, proliferation and migration of endothelial cells of the EA.hy926 cell line. Endothelial cells were cultured with MVs derived from cells of the NK-92 cell line after their stimulation with IL-1β. Using flow cytometry, we evaluated changes in the expression of endothelial cell surface molecules and endothelial cell death. We evaluated the effect of MVs derived from stimulated NK cells on the proliferative and migratory activity of endothelial cells, as well as the activation of caspase-3 and caspase-9 therein. It was established that the incubation of endothelial cells with MVs derived from cells of the NK-92 cell line stimulated with IL-1β and with MVs derived from unstimulated NK cells, leads to the decrease in the proliferative activity of endothelial cells, appearance of the pan leukocyte marker CD45 on them, caspase-3 activation and partial endothelial cell death, and reduced CD105 expression. However, compared with MVs derived from unstimulated NK cells, a more pronounced effect of MVs derived from cells of the NK-92 cell line stimulated with IL-1β was found in relation to the decrease in the endothelial cell migratory activity and the intensity of the CD54 molecule expression on them. The functional activity of MVs is therefore mediated by the conditions they are produced under, as well as their internal contents.

## 1. Introduction

Intercellular communications underlie various physiological and pathological processes in the body. It is now believed that interactions between cells can be mediated by extracellular vesicles [1]. Extracellular vesicles are a heterogeneous population of membrane vesicles produced by cells during their vital activity [2,3,4,5]. Extracellular vesicles are found in almost all human biological fluids, which has aroused a close interest in their comprehensive study in the scientific community [2,6,7,8]. One of the types of extracellular vesicles are MVs, which are subcellular structures ranging in size from 100 (150) to 1000 nm, formed by budding (blebbing) of the plasma membrane (PM) of the parent cell [9,10].

MVs represent a heterogeneous population of extracellular vesicles [11], whose functional activity is determined largely by their internal contents and molecules expressed on their surface [12]. According to the literature sources, MVs involves various proteins (matrix metalloproteinases [13], glycoproteins [14], integrins [15], signalling proteins, metabolic enzymes, cytoskeletal proteins [16], mitochondrial proteins [9] and ribosomal and centrosomal proteins [17]) and nucleic acids (mRNA [18,19], microRNA [1] and DNA [20]). It is believed that the internal composition of MVs largely depends on the type of source cells [12,21] and their microenvironment [9], as well as on pathological conditions [22]. Currently, the most characterised MVs are those of platelets, monocytes and endothelial cells [23]. Researches on the properties of tumour MVs are of great interest [10]. MVs of leukocyte origin are the least studied population of MVs. This may be because they constitute only a minor fraction of MVs in the bloodstream under normal physiological conditions [8]. In pathological processes, the level of leukocyte-derived MVs in blood increases dramatically, so that they are considered to be markers for development of various diseases [24].

MVs derived from NK cells are of particular interest for study. NK cells are involved in the realisation of both innate and adaptive immune responses [25], in various physiological and pathological processes, such as remodelling of the uterine spiral arteries during pregnancy, placental angiogenesis, inflammation, tumour formation, endometriosis and miscarriage [26,27,28,29,30]. Current literature sources contain a small amount of data on the properties and functional characteristics of MVs derived from NK cells. Most studies of extracellular vesicles derived from NK cells are associated with the study of their cytotoxic potential and the search for molecules mediating it [31,32].

The endothelium is one of the most convenient targets for studying MVs derived from NK cells, since in vivo, when realising their functional activity, NK cells interact with endothelial cells everywhere (the process of leukocyte transendothelial migration) [25,33]. Interactions between NK cells and endothelial cells further underlie many physiological and pathological processes, such as angiogenesis, inflammation, tumour formation and endometriosis [26,34,35,36]. Endothelial cells are characterised by the expression of many receptors, including PECAM-1 (CD31), integrin β1 (CD29), CD34, CD119 (IFNγ-receptor), VE-cadherin (CD144), VEGFR1, ICAM-1 (CD54), CD105 and glycoproteins [37,38,39,40,41]. Changes in the phenotypic profile of endothelial cells may affect the functional state of cells. It is presumed that MVs may be one of the mechanisms for realising the functional activity of parent cells. In this regard, the study of MVs derived from NK cells and their interactions with the endothelium will reveal new aspects of communication between endothelial cells and NK cells, expand knowledge about this type of MVs and make a significant contribution to understanding the mechanisms of the physiological and pathological processes described above.

Previously, we showed the effect of MVs derived from NK cells on the functional activity of endothelial cells under physiological conditions [42]. A significant difference in the proteomic profiles of cells of the NK-92 cell line and MVs derived from them was established. We also found that the contact of endothelial cells with MVs derived from cells of the NK-92 cell line led to partial endothelial cell death, expression of granzyme B and Erk, as well as to activation of caspase-9 and caspase-3 in them. It also led to the appearance of the CD45 pan leukocyte marker on endothelial cells, reduced expression of CD105, more pronounced expression of CD34 and CD54 and inhibition of endothelial cell functional activity. The literature sources contain evidence that microenvironment and activation of MV source cells affect the composition and functional characteristics of MVs [9]. In this regard, we aimed to study the role of MVs derived from NK cells stimulated with IL-1β in changing the functional and phenotypic characteristics of endothelial cells. To assess the effects of the microenvironment and pathological condition on the change in the characteristics of NK cells and their MVs, we used the proinflammatory cytokine IL-1β, which promotes NK cell activation [43].

## 2. Results

### 2.1. Effects of MVs Derived from NK Cells of the NK-92 Cell Line Stimulated with IL-1β on the Proliferative Activity of Endothelial Cells of the EA.hy926 Cell Line

**Figure 1:**
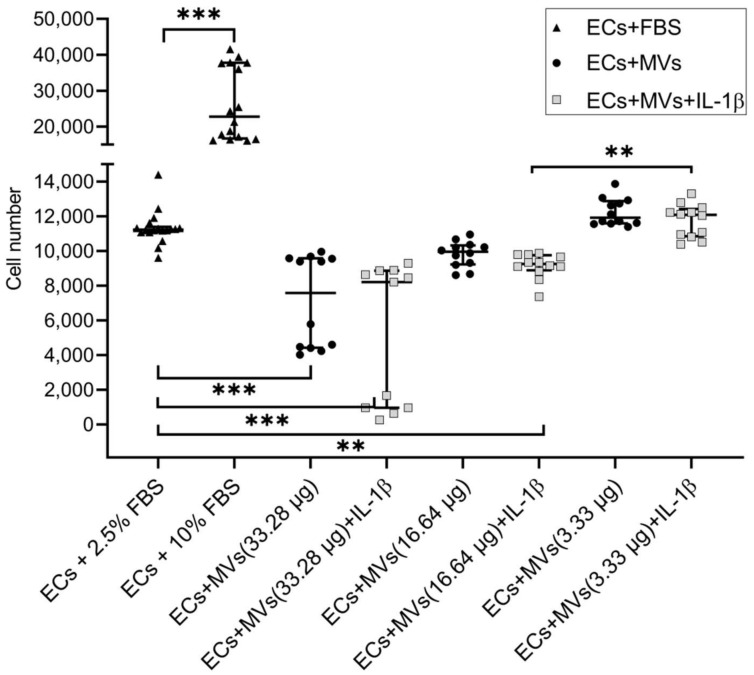
Effects of MVs Derived from Unstimulated Cells and Cells Stimulated with IL-1β on the Proliferative Activity of Endothelial Cells of the EA.hy926 Cell Line. 10% FBS (foetal bovine serum) —positive control. 33.23 μg, 16.64 μg, 3.33 μg—total protein concentration in MVs derived from unstimulated cells and cells stimulated with IL-1β per 100 µL of medium. Significance of differences: ** *p* < 0.01; *** *p* < 0.001.

We have previously established the effect of MVs derived from unstimulated NK cells of the NK-92 cell line on the proliferative activity of endothelial cells of the EA.hy926 cell line (the results are given in the article [42]). The culturing of endothelial cells with MVs derived from NK cells of the NK-92 cell line stimulated with IL-1β, containing a total protein content of 33.28 μg/100 μL and 16.64 μg/100 μL, resulted in the dose-dependent decrease in the proliferative activity of endothelial cells compared with the baseline (Figure 1). At the same time, there was no difference between the effects of MVs derived from unstimulated cells and cells stimulated with IL-1β on the proliferative activity of endothelial cells of the EA.hy926 cell line.

### 2.2. Effects of MVs Derived from NK Cells of the NK-92 Cell Line Stimulated with IL-1β on the Migratory Activity of Endothelial Cells of the EA.hy926 Cell Line

**Figure 2:**
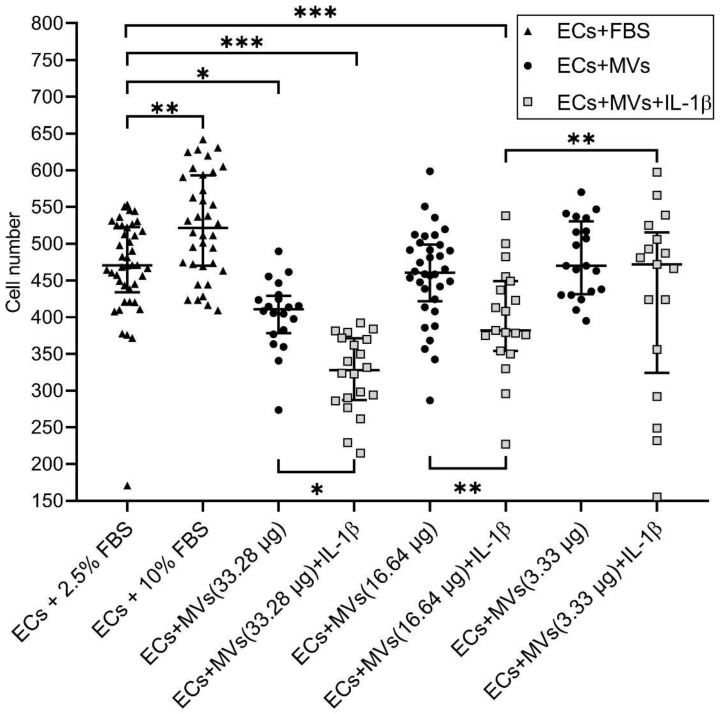
Effects of MVs Derived from Unstimulated Cells and Cells Stimulated with IL-1β on the Migratory Activity of Endothelial Cells of the EA.hy926 Cell Line (number of cells). 10% FBS—positive control. 33.23 μg, 16.64 μg, 3.33 μg—total protein concentration in MVs derived from unstimulated cells and cells stimulated with IL-1β per 100 µL of medium. Statistical significance between groups: * *p* < 0.05, ** *p* < 0.01, *** *p* < 0.001.

**Figure 3:**
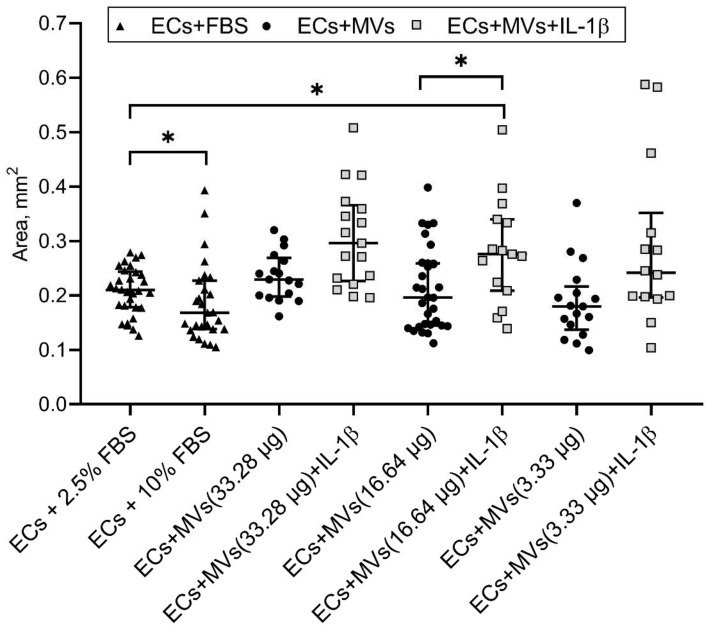
Effects of MVs Derived from Unstimulated Cells and Cells of the NK-92 Cell Line Stimulated with IL-1β on the Migratory Activity of Endothelial Cells of the EA.hy926 Cell Line (residual area after migration). 10% FBS—positive control. 33.23 μg, 16.64 μg, 3.33 μg—total protein concentration in MVs derived from unstimulated cells and cells stimulated with IL-1β per 100 µL of medium. Statistical significance between groups: * *p* < 0.05.

We have previously established the effect of MVs derived from unstimulated NK cells of the NK-92 cell line on the migratory activity of endothelial cells of the EA.hy926 cell line (the results are given in the article [42]). The incubation of endothelial cells with MVs derived from cells of the NK-92 cell line stimulated with IL-1β, containing a total protein content of 16.64 µg/100 µL and 33.28 µg/100 µL, resulted in the dose-dependent decrease in the number of migrated endothelial cells compared with the baseline and compared with the culturing of endothelial cells with MVs derived from unstimulated cells of the NK-92 cell line with the same protein content (Figure 2, Figure S2). It was also established that the culturing of endothelial cells with MVs derived from cells of the NK-92 cell line stimulated with IL-1β, containing a total protein content of 16.64 µg/100 µL, resulted in the increase in the residual area after endothelial cell migration, both compared with the baseline and compared with the culturing of endothelial cells with MVs derived from unstimulated cells of the NK-92 cell line with the same protein content (Figure 3).

### 2.3. Effects of MVs Derived from NK-92 Cells Stimulated with IL-1β on the Phenotype of ECs of the Ea.hy926 Cell Line

**Figure 4:**
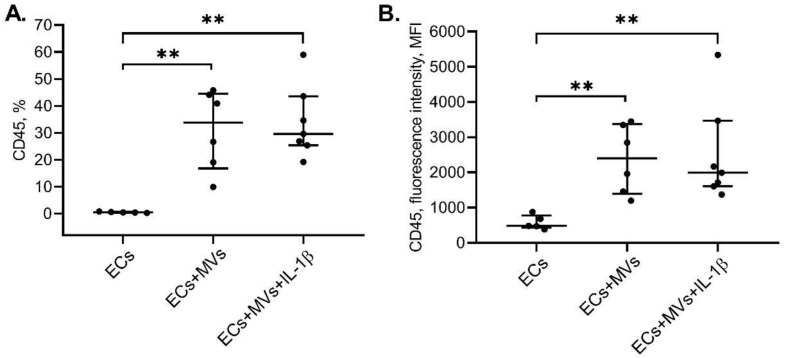
Appearance of the CD45 Molecule Expression by Cells of the EA.hy926 Cell Line after their Incubation with MVs Derived from MVs Derived from Unstimulated Cells (MVs) and Cells of the NK-92 Cell Line Stimulated with IL-1β (MVs + IL-1β). MVs derived from unstimulated cells and cells of the NK-92 cell line stimulated with IL-1β were used at a concentration of 33.28 µg of total protein in 100 μL of medium. Data are presented as “median {lower quartile; upper quartile}”. (**A**)—the relative number of endothelial cells expressing the CD45 molecule; (**B**)—the intensity of the CD45 molecule expression by endothelial cells. MFI—mean fluorescence intensity. Statistical significance between groups: ** *p* < 0.01.

Previously, the effect of MVs derived from unstimulated cells of the NK-92 cell line on the phenotype of endothelial cells of the Ea.hy926 cell line was shown [42]. Following the incubation of endothelial cells of the EA.hy926 cell line with MVs derived from cells of the NK-92 cell line stimulated with IL-1β, the presence of endothelial cells of the EA.hy926 cell line with the CD45+ phenotype was revealed. The intensity of CD45 expression by endothelial cells of the EA.hy926 cell line was also increased after endothelial cell incubation with MVs derived from cells of the NK-92 cell line stimulated with IL-1β compared with unstimulated endothelial cells of the EA.hy926 cell line (Figure 4).

**Figure 5:**
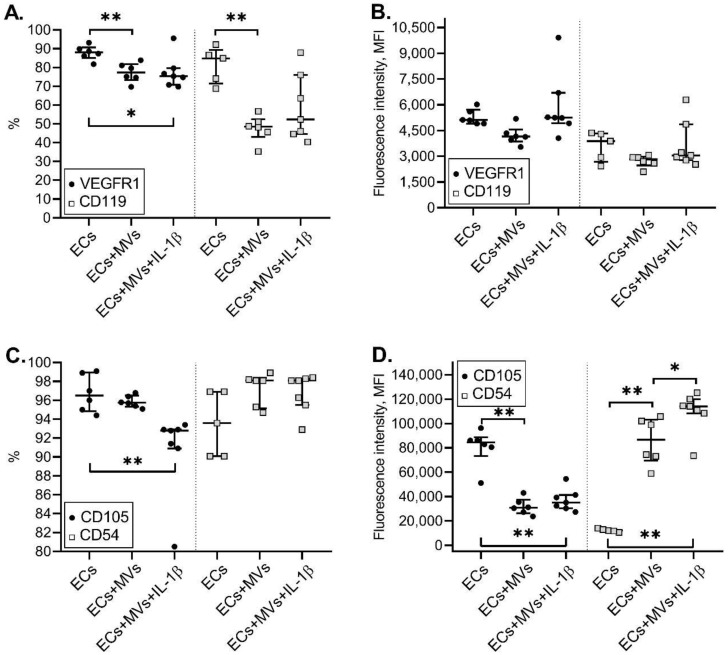
Effects of MVs Derived from Unstimulated Cells (MVs) and Cells of the NK-92 Cell Line Stimulated with IL-1β (MVs + IL-1β) on the Appearance of Expression of Surface Receptors by Endothelial Cells of the EA.hy926 Cell Line. MVs derived from unstimulated cells and cells of the NK-92 cell line stimulated with IL-1β were used at a concentration of 33.28 µg of total protein in 100 μL of medium. Data are presented as “median {lower quartile; upper quartile}”. The relative number of endothelial cells expressing receptors: (**A**)—VEGFR1, CD119; (**C**)—CD54, CD105. The intensity of the molecule expression by endothelial cells: (**B**)—VEGFR1, CD119; (**D**)–CD54, CD105. MFI—mean fluorescence intensity. Significance of differences: * *p* < 0.05; ** *p* < 0.01.

The culturing of endothelial cells with MVs derived from NK cells stimulated with IL-1β resulted in the decreased number of endothelial cells with the VEGFR1+ phenotype compared with the number of endothelial cells under unstimulated incubation conditions. At the same time, the intensity of this receptor expression was also reduced after the treatment of endothelial cells with MVs derived from unstimulated NK cells and did not differ from that after the treatment of endothelial cells with MVs derived from NK cells treated with IL-1ß. The incubation of endothelial cells of the EA.hy926 cell line with MVs derived from NK cells stimulated with IL-1β, resulted in the decrease in the intensity of the CD105 molecule expression by endothelial cells compared with the intensity of CD105 expression by unstimulated endothelial cells (Figure 5). However, although there is no difference in the number of CD54+ endothelial cells treated with MVs derived from NK cells stimulated with IL-1β, there was an increase in the intensity of the CD54 molecule expression by endothelial cells compared with both unstimulated endothelial cells and endothelial cells incubated with MVs derived from unstimulated cells of the NK-92 cell line (Figure 5).

### 2.4. Effects of MVs Derived from Cells of the NK-92 Cell Line Stimulated with IL-1β on Caspase-3 and Caspase-9 Activity in Endothelial Cells of the EA.hy926 Cell Line

**Figure 6:**
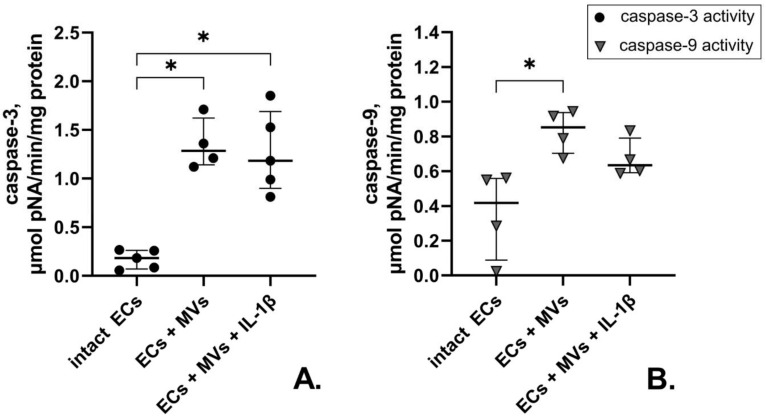
Enzymatic Activity of Caspase-9 and Caspase-3 in Endothelial Cells of the EA.hy926 Cell Line after their Culturing with MVs derived from Unstimulated Cells and Cells of the NK-92 Cell Line Stimulated with IL-1β. MVs derived from unstimulated cells and cells of the NK-92 cell line stimulated with IL-1β were used at a concentration of 33.28 µg of total protein in 100 μL of medium. Data are presented as “median {lower quartile; upper quartile}”. (**A**)—Enzymatic Activity of caspase-3 activity in endothelial cells of the EA.hy926 cell line; (**B**)—Enzymatic Activity of caspase-9 activity in endothelial cells of the EA.hy926 cell line Statistical significance: * *p* < 0.05.

We have previously shown the increased enzymatic activity of caspase-3 and caspase-9 in cells of the EA.hy926 cell line collected after their culturing with MVs derived from unstimulated cells of the NK-92 cell line, compared with unstimulated endothelial cells [42]. At the same time, the culturing of endothelial cells of the EA.hy926 cell line with MVs derived from cells of the NK-92 cell line stimulated with IL-1β resulted in the increased enzymatic activity of caspase-3 only, which was expressed in the increased amount of the enzymatic reaction product. Meanwhile, there was no difference between the effects of MVs derived from unstimulated cells and cells stimulated with IL-1β on the caspase-3 activity in endothelial cells of the EA.hy926 cell line (Figure 6).

## 3. Discussion

The study of MV involvement in the pathogenesis of various diseases is a topical area of modern biology. However, the study of MVs derived from biological fluids is associated with methodological difficulties and a limited amount of the collected material [44,45]. One of the available approaches is the use of cell lines. The choice of cells of the NK-92 cell line is due on the one hand to the lack of data on MVs produced by them, and on the other hand, it is associated with the promising use of cells of this line for therapeutic purposes [46]. Previously, we assessed MVs produced by unstimulated cells of the NK-92 cell line. Our findings indicated a significant difference in proteomic profiles of cells of the NK-92 cell line and MVs produced by them. We also showed that the contact between endothelial cells and unstimulated MVs derived from cells of the NK-92 cell line induced apoptosis activation in endothelial cells. We also showed the dose-dependent effect of MVs: at a concentration 10 times lower than that which causes endothelial cell death, MVs promoted the increase in endothelial cell proliferation. As the activation of MV source cells affects the composition and functional characteristics of MVs [9], this study involved a cultural model of interaction between endothelial cells of the EA.hy926 cell line and MVs derived from cells of the NK-92 cell line collected after NK cell stimulation with IL-β in order to reproduce the inflammatory conditions.

Using a flow cytometer, we found that the death of endothelial cells of the EA.hy926 cell line after their culturing with MVs derived from cells of the NK-92 cell line stimulated with IL-β was at the same level as with MVs derived from unstimulated cells of the NK-92 cell line [42], and amounted to 29.9%, while the spontaneous endothelial cell death amounted to 6%.

Using culture methods, we found that MVs derived from NK cells of the NK-92 cell line stimulated with IL-β suppressed the migratory and proliferative activity of endothelial cells in a dose-dependent manner, while the introduction of IL-1β into the system enhanced the inhibitory effect of MVs on the endothelial cell migratory activity. As in the case of unstimulated MVs, endothelial cell culturing with MVs derived from cells of the NK-92 cell line stimulated with IL-β resulted in the CD45 molecule transfer to endothelial cells and in the decrease in the intensity of CD105 expression by endothelial cells.

According to the literature sources, MVs and cells can interact in different ways. MVs can be internalised and transfer their contents to cells through a simple fusion of plasma and endosomal membranes, clathrin- and caveolin-mediated endocytosis, lipid raft-dependent endocytosis, phagocytosis and micropinocytosis, as well as through ligand-receptor interactions with the target cell and the release of MV contents into the extracellular space in the immediate vicinity of the cell [2,12,47,48]. The internalisation of extracellular vesicles, including MVs, depends largely on the type of proteins and glycoproteins that are expressed both on MVs themselves and on recipient cells [49].

Early on, when studying the phenotypic and functional characteristics of MVs derived from cells of the NK-92 cell line using a Western blot method, we showed that MVs derived from unstimulated cells and cells of the NK-92 cell line stimulated with IL-1β contained cytotoxic proteins granzyme B and perforin. Parent cell stimulation with IL-1β did not result in the increase in the amount of total protein in MVs, but caused the increase in perforin expression and the decrease in granzyme B expression in them [50]. Moreover, by using the flow cytometry and Western blot method, we showed that MVs derived from unstimulated cells of the NK-92 cell line were able to transfer their contents, including fluorescently stained proteins and granzyme B, into endothelial cells of the EA.hy926 cell line [42]. When assessing the effect of MVs produced by unstimulated NK cells, we showed a decrease in the expression of CD105, CD119 and VEGFR1 by endothelial cells, and an increase in the expression of CD34 and CD54 receptors [42]. MVs derived from cells of the NK-92 cell line stimulated with IL-1β also interacted with endothelial cells, changed their functions and properties, apparently by transferring their contents and/or embedding in the cell PM. This is further supported by similar results regarding endothelial cell viability and the decrease in the number of cells expressing VEGFR1, changes in endothelial cell proliferative and migratory activity, CD45 membrane protein transfer to them and changes in their phenotype—the decrease in the intensity of the CD105 molecule expression [42].

No increase in endothelial cell death along with simultaneous increase in inhibitory properties against proliferative and migratory activity of endothelial cells after their culturing with MVs derived from cells of the NK-92 cell line stimulated with IL-1β, compared with MVs derived from unstimulated cells, means that MVs derived from stimulated cells, such as MVs derived from unstimulated cells [42], could cause the transfer of granzyme B and perforin to endothelial cells and initiate endothelial cell apoptosis. However, the established differences in cytotoxic protein expression in MVs derived from unstimulated and stimulated cells suggest that MVs derived from stimulated and unstimulated cells of the NK-92 cell line could influence endothelial cells via different mechanisms, or MVs derived from stimulated cells have additional ways to affect cells due to differences in their content. This assumption is borne out by other effects of MVs derived from cells of the NK-92 cell line stimulated with IL-1β on the endothelium found in this study. Using flow cytometry, we established the increase in the CD54 molecule expression by endothelial cells after their incubation with MVs derived from cells of the NK-92 cell line stimulated with IL-1β compared with unstimulated endothelial cells and endothelial cell cultured with unstimulated NK cells. In addition, it was found that endothelial cell incubation with MVs derived from cells of the NK-92 cell line stimulated with IL-1β, unlike incubation with MVs derived from unstimulated cells [42], did not lead to the increase in the enzymatic activity of initiator caspase-9; while in both cases, activation of effector caspase-3 was observed.

The increase in the intensity of the CD54 adhesion molecule expression that we found is evidence in favour of endothelial cell activation, which is consistent with the literature data [51,52,53]. We have previously found that 20–39% of MVs expressed the CD54 molecule, while cells of the NK-92 cell line stimulation with IL-β did not affect the change in the expression of this molecule by MVs [50]. In this regard, the increase in the intensity of CD54 expression by endothelial cells after incubation with MVs derived from unstimulated cells and cells of the NK-92 line stimulated with IL-1β indicates that MVs, internalising in endothelial cells, transmit an activation signal to them, and do not simply embed in the PM, and thus transfer their surface molecules to endothelial cells. The increase in the intensity of the ICAM-1 molecule expression after endothelial cell incubation with MVs derived from stimulated cells, compared with MVs derived from unstimulated cells, confirms the fact that the source cell activation changes the MV contents, which in turn affects the effect caused by MVs on target cells. MVs derived from NK cells apparently contain microRNAs, cytokines, growth factors and other BAS, which, upon entering endothelial cells from MVs, could cause their activation. The addition of IL-1β to NK cells could stimulate their metabolic activity and production of biologically active factors, microRNAs, which, being transported to endothelial cells by means of MVs, contributed to endothelial cell activation and expression of adhesive molecules on them. This fact is consistent with the literature data: for exosomes of NK cells isolated from peripheral blood mononuclears, it was shown that mononuclear activation did not lead to a change in their protein content, while the functional activity of stimulated and unstimulated exosomes differed [54]. There is also evidence that the activation of the same cells by different agents leads to a change in the characteristics of MVs they release [16]. MVs derived from NK cells therefore act as activating agents capable of inducing the adhesive molecule expression by endothelial cells. MV functional activity can apparently be due to release of biologically active factors from them in the immediate vicinity of the target cell, as well as internalisation into the cell and transfer of activating mediators to it and the incorporation of receptors expressed by them into PM, which was shown for the CD45 molecule.

The effect of MVs derived from cells of the NK-92 cell line stimulated with IL-1β on the viability and functional activity of endothelial cells is apparently associated not only with granzyme B transfer to them, but also with the altered MV contents. The decrease in granzyme B expression in MVs derived from stimulated cells, did not reduce the cell death, yet caused the increased inhibition of endothelial cells’ migratory and proliferative properties. The observed effect of MVs derived from stimulated cells may be associated with the activation of parent cells. NK cells are a source of various cytokines, including IFNγ, the secretion of which increases upon NK cell activation. According to the literature sources, the pro-inflammatory cytokine IFNγ has an antiangiogenic effect and reduces endothelial cell viability [3]. MVs derived from stimulated NK cells could therefore reduce endothelial cell proliferation and migration by releasing IFNγ in the immediate vicinity of the target cell. This assumption is consistent with the result of the increase in the residual area after migration of endothelial cells incubated with MVs derived from cells of the NK-92 cell line stimulated with IL-1β.

The observed increased enzymatic activity of caspase-3 with no increase in the enzymatic activity of caspase-9 in endothelial cells, after their culturing with MVs derived from stimulated NK cells, suggests that MVs derived from stimulated cells can cause activation of other initiator caspases and/or direct activation of caspase-3 with granzyme B [55]. There is a possibility that MVs can initiate an exogenous pathway of apoptosis. According to the literature sources, cells of the EA.hy926 cell line express TRAIL receptors on their surface [56], which are involved in the induction of the external pathway of apoptosis. MVs derived from both unstimulated and stimulated NK cells caused endothelial cell activation, which is confirmed by the increased intensity of CD54 expression, which means that they can induce the expression of TRAIL receptors on endothelial cells. NK cells are the source of TNFa, their MVs can contain this cytokine and release it in close proximity to endothelial cells. Regarding this connection, more research is needed to elucidate the mechanisms of apoptosis mediated by MVs derived from NK cells.

## 4. Materials and Methods

### 4.1. Cell Lines

NK cells of the NK-92 line (ATCC, Virginia, USA) were used as a source of MVs [57]. Cells of the NK-92 cell line display the main phenotype and functional characteristics of NK cells [57,58]. Endothelial cells of the EA.hy926 cell line (ATCC, Virginia, USA) were used as target cells. Cells of the EA.hy926 line reproduce the main morphological, phenotypic and functional characteristics of the endothelium [59,60,61].

All cell lines were cultured following the manufacturer’s instructions (ATCC, Virginia, USA) in a humid atmosphere at 37 °C, 5% CO_2_. Cell viability was assessed using Trypan blue solution (Sigma–Aldrich Chem. Co., St. Louis, MO, USA) and was at least 96%. All experiments, including cell culturing, were performed at 37 °C, 5% CO_2_.

Human recombinant cytokine IL-1β in the concentration of 1000 ng/mL was used as an inducer (specific activity 1 IU = 0.01 ng) (“Betaleikin”, Research Institute of Highly Pure Biopreparations, St. Petersburg, Russia). Culturing in the complete cell culture medium without inducer served as the control condition.

### 4.2. MV Isolation

Cells of the NK-92 cell line were cultured in 75 cm^2^ flasks (BD, Franklin Lakes, NJ, USA) in complete growth medium α-MEME. The day before MV isolation, the culture medium was completely changed, volume was brought to 40 mL with the cell concentration of 4 × 10^5^ in 1 mL. Some of the cells were stimulated with IL-1β (1000 ng/mL). Cell viability was assessed the day after culture initiation using Trypan blue solution and was at least 96%. As there is no single standard for isolation and characterisation of MVs, various methodological approaches were used that allow MV fractions to be obtained that differ in purity and enrichment level [62,63]. We isolated MVs derived from cells of the NK-92 cell line using a modified method of differential centrifugation [6,64] in Hanks′ solution without CaCl_2_ and MgCl_2_ (Sigma–Aldrich Chem. Co., St. Louis, MO, USA). The culture medium from flasks was centrifuged at 200× *g* to enable settling and removal of cells (22 °C, 10 min). The resulting supernatants were subsequently centrifuged at 500× *g* (4 °C, 10 min), 9900× *g* (4 °C, 11 min) and 19,800× *g* (4 °C, 20 min). Following the last centrifugation, the MV precipitate was washed twice with Hanks′ solution without CaCl_2_ and MgCl_2_ and centrifuged again at 19,800× *g* (4 °C, 20 min), then the supernatant was removed. This procedure provides for MVs of 100–1000 nm in diameter to be isolated with sufficient purity and minimal loss of biomaterial (Figure S1). MVs can be successively separated from coarse particles of cellular debris and apoptotic bodies, as well as from exosomes [50,51]. All solutions and media for MV isolation, including Hanks’ solution without CaCl_2_ and MgCl_2_, FBS (foetal bovine serum) (BioloT, St. Petersburg, Russia) and horse serum (BioloT, St. Petersburg, Russia) were pre-filtered through an ultrafilter (Corning, Kaiserslautern, Germany) with a pore diameter of 0.2 μm [45].

### 4.3. MV Analysis by Dynamic Light Scattering

To control the size of isolated MVs, their granulometric analysis was carried out by the dynamic light scattering method using Zetasizer NanoZS (Malvern Instruments, Malvern, UK) with the range of particle measurements from 0.3 nm to 10 μM. The MV diameter was calculated using the Zetasizer Software 7.11 (Malvern Instruments, Malvern, UK). The size of MVs derived from NK cells of the NK-92 cell line ranged from 210 nm to 490 nm, and the peak of the MV quantity distribution was 315 nm. These data complied with our previous work [65,66] and that of other research groups that ascertained the size of MVs derived from various cells [6,45,67]. The addition of IL-1β did not change the average size of MVs. They stayed in the same size range as MVs produced by inactivated NK cells. The reproducibility of the MV granulometric analysis results obtained in our laboratory at different times has led us to recommend laser correlation analysis as a benchmark for assessing the isolation purity of these extracellular objects.

### 4.4. Analysis of Total Protein Content of Cell and MV Lysates

The protein content of cell and MV lysates was determined by the Bradford assay [68] using a NanoDrop One spectrophotometer (Thermo Scientific, Waltham, MA, USA). The protein content in NK cells did not differ in different culturing conditions (for unstimulated cells: 61.2 ± 6.3 μg/10^6^ cells, for cells pre-treated with IL-1β (as described above): 66.3 ± 6.0 μg/10^6^ cells). MVs derived from unstimulated cells and those derived from cells treated with IL-1ß also did not differ in protein content and amounted to 4.6 ± 0.6 and 2.6 ± 0.4 µg per 10^6^ source cells, respectively.

### 4.5. Preparation of MV Dilutions

Based on the protein content in MVs derived from cells of the NK-92 line stimulated with IL-1β, as well as in MVs derived from unstimulated cells, MV dilutions were prepared: 33.28 μg/100 μL, 16.64 μg/100 μL, 3.33 μg/100 μL of culture medium. We used these dilutions earlier in experiments to evaluate the effects of MV derived from unstimulated cells of the NK-92 cell line on the phenotypic and functional characteristics of endothelial cells of the EA.hy926 cell line [42]. In experiments assessing the migratory and proliferative activity of endothelial cells treated with MVs derived from NK cells, we used three variants of MVs dilution. For further evaluation of changes in the phenotypic profile of endothelial cells and changes in the content of caspases in endothelial cells under the influence of MVs, the maximum concentration of MVs (33.28 µg/100 µL) was used.

### 4.6. Evaluation of the Effect of MVs Derived from Cells of the NK-92 Cell Line on the Migratory Activity of Endothelial Cells of the EA.hy926 Cell Line

The day before the experiment, endothelial cells were added to wells of a 96-well flat bottom plate (Sarstedt, Nümbrecht, Germany) (3.5 × 10^4^ cells per well in 0.1 mL of medium, 10% FBS) and cultured for 24 h. The monolayer was then disrupted by partial cell scraping. For this purpose, we used a 200 μL pipette tip to draw a vertical straight line in the middle of each well from edge to edge and then washed the line with warm Hanks’ solution (BioloT, St. Petersburg, Russia). The width of the obtained line of the disrupted monolayer was photographed.

Following this, the medium for cells of the EA.hy926 cell line was replaced with dilutions of MVs derived from unstimulated cells and cells of the NK-92 cell line stimulated with IL-1β that were prepared using the endothelial cell medium containing 2.5% FBS. The cells were then cultured for 24 h. Then, endothelial cells were incubated for 10 min with 100 μL of 0.2% crystal violet solution (Sigma–Aldrich Chem. Co., St. Louis, MO, USA) containing 5% methanol. After that, the plate was washed with distilled water and dried. Three fields of view were photographed in each well. Analysis of the obtained data was carried out using MarkMigration software (St. Peterburg, Russia) [69], which automatically considers the residual area of the disrupted monolayer line after migration. In each photograph, two parallel lines of the disrupted monolayer (mm^2^) were run and the number of cells that migrated to the zone of the disrupted monolayer line was specified. Change in the cell migratory activity was assessed by evaluating the change in the number of cells that migrated during the experiment compared with controls. It was also assessed by evaluating the change in the area of the disrupted monolayer line after cell migration in a well compared with controls (Figure S2).

Experiments determining endothelial cell migratory activity in the presence of MVs were performed three times. Each MV concentration was analysed four times. Culture medium containing 2.5% FBS was used as a control sample. Culturing in the medium containing 10% FBS was used as a positive control. The area of the initial line when scraping the monolayer was 0.53 {0.48; 0.53} mm^2^. No cells in the zone of the disrupted monolayer were revealed. We noted the increase in the number (470.5 (438.3, 522.3), *p* < 0.001) of migrated endothelial cells and the decrease in the area (0.21 (0.18, 0.24), *p* < 0.001) mm^2^ of the disrupted monolayer line after the cell migration in the presence of 2.5% FBS. The increase in the FBS concentration of the culture medium to 10% (positive control) caused the increase in the number of migrated endothelial cells (521.3 (470.8, 592.3), *p* < 0.01) and the decrease in the residual area of the disrupted monolayer line after cell migration (0.16 (0.14, 0.22), *p* < 0.05) mm^2^. Thus, data of control samples (2.5% FBS and 10% FBS shown as median (interquartile range)) about number of migrated endothelial cells and area after cell migration allow to conclude that cells of the EA.hy926 cell line responded to a higher FBS concentration with the increased migratory activity, which is consistent with results described previously [70,71]. This allows the evaluation of changes in endothelial cell migratory activity in the presence of MVs derived from cells of the NK-92 cell line.

### 4.7. Evaluation of the Effect of MVs Derived from Cells of the NK-92 Cell Line on the Proliferative Activity of Endothelial Cells of the EA.Hy92 Cell Line

The day before the experiment, endothelial cells were added to wells of a 96-well flat bottom plate (2.5 × 10^3^ cells per well in 0.1 mL of medium, 10% FBS) and cultured for 24 h. The medium was then replaced with dilutions of MVs derived from unstimulated cells and cells of the NK-92 cell line stimulated with IL-1β that were prepared using the endothelial cell medium containing 2.5% FBS. Then the cells were cultured for 72 h. Medium containing 2.5% FBS was used as a control sample. Medium containing 10% FBS was used as a positive control sample. Then, endothelial cells were stained with 0.2% crystal violet solution containing 5% methanol, for which the dye was added to each well in the volume of 100 μL per well and incubated for 10 min. After staining, wells were washed with distilled water four times (Figure S3). The plate was dried and the dye was extracted with 50% acetic acid solution. The optical density was calculated using a Labsystems Microplate Reader (BioTek, Winooski, VT, USA) at a wavelength of 540 nm (cut-off 620 nm). The resulting optical densities were converted to the cell number using a titration curve. The results were represented as the cell numbers. The change in the proliferation level was assessed by the change in the sample optical density and cell number compared with the incubation in the culture medium for endothelial cells with addition of 2.5% FBS without MVs. When culturing endothelial cells with 10% FBS (positive control), stimulation of endothelial cell proliferative activity was observed compared with endothelial cells cultured with 2.5% FBS (with 10% FBS—11,169.1 (10,612.69, 11,362.76) cells; with 2.5% FBS—38,577.2 (16,583.2, 39,818.4) cells, *p* < 0.001). Experiments were carried out three times. MV concentrations were analysed four times.

### 4.8. Evaluation of the Effect of MVs Derived from Cells of the NK-92 Cell Line on the Phenotype of Endothelial Cells of the Ea.hy926 Cell Line

The day before the experiment, endothelial cells were added to wells of a 96-well flat bottom plate (3.5 × 10^4^ cells per well in 100 μL of medium) and cultured for 24 h. Next, the medium was removed from the plate along with the endothelial cell monolayer. MVs derived from unstimulated cells and cells of the NK-92 cell line stimulated with IL-1β were then added at a concentration of 33.28 µg of total protein in 100 μL of medium (in three repetitions). We used the concentration of MVs amounting to 33.28 µg of total protein in 100 µL of medium, since at the stage of assessing the migratory and proliferative activity of endothelial cells, the most pronounced effects were found in the case of treatment of cells with MVs at this concentration. Unstimulated endothelial cells were used as controls. Endothelial cells incubated with 10 ng/mL phorbol-12-myristate 13-acetate (Sigma–Aldrich Chem. Co., St. Louis, MO, USA) were used as positive controls. One day later, endothelial cells were washed three times with warm Hanks’ solution and removed from the plate with Versene solution (BioloT, St. Petersburg, Russia). To wash the cells from Versene solution, Hanks’ solution was used. To control the survivability, endothelial cells were stained with 7-AAD dye (Biolegend, San Diego, CA, USA), and the cell death rate was assessed using the FACS Canto II flow cytometer by 7-AAD inclusion, as described above [72,73]. The pool of nonviable endothelial cells after culturing with MVs, derived from cells of the NK-92 cell line stimulated with IL-1β, was a median (interquartile range) of 29.9% (26.3, 54.5). Viability experiments were repeated four times. Following the incubation with MVs, endothelial cells were treated with monoclonal antibodies to CD119, CD54, CD45 (Becton Dickinson, Franklin Lakes, NJ, USA), VEGFR1 and CD105 (R&D Systems, Minneapolis, MN, USA), as well as with isotypic antibodies according to the manufacturer’s instructions. The choice of receptors was made based on the results of experiments assessing the phenotype of endothelial cells after treatment with MVs derived from unstimulated NK cells. Fluorescence was assessed using a FACS Canto II flow cytofluorimeter (Becton Dickinson, Franklin Lakes, NJ, USA) (the gating strategy is described in a previously published article [42]). Analysis of the receptor expression by endothelial cells was repeated four times.

### 4.9. Evaluation of the Eeffect of MVs Derived from Cells of the NK-92 Cell Line on Caspase Activity in ECs of the Ea.Hy926 Cell Line

The activity of caspase-3 and caspase-9 was determined in lysates of endothelial cells of the EA.hy926 cell line treated with MVs derived from unstimulated cells and cells of the NK-92 cell line stimulated with IL-1β. The protein concentration in the lysates of cells of the EA.hy926 cell line was measured by the Bradford assay using a NanoDrop OneC instrument.

The caspase activity was determined using a spectrophotometric method and specific substrates; a reaction buffer containing 20 mM HEPES (Sigma–Aldrich Chem. Co., St. Louis, MO, USA), 0.1% CHAPS (Bio-Rad, Hercules, CA, USA), 2 mM EDTA (Sigma–Aldrich Chem. Co., St. Louis, MO, USA), 5 mM DTT (Bio-Rad, Hercules, CA, USA), pH 7.4 was also used. The substrates used were 0.2 mM solutions of synthetic peptides Ac-DEVD-pNA for caspase-3 (Enzo Life Sciences, New York, NY, USA) and Ac-LEHD-pNA for caspase-9 (Enzo Life Sciences, New York, NY, USA), respectively. For experiments assessing the activity of caspases, the concentration of MVs amounting to 33.28 µg/100 µL of medium was selected based on the previous results. The increase in the pNA reaction product was assessed by colorimetric analysis at a wavelength of 405 nm for 150 min. The caspase activity was then assessed using the formula (ODt−OD0)/(t × ε × c), where t is reaction time in minutes, OD0 is absorption measured before adding the substrate, ODt is absorption measured t minutes after adding the substrate, ε is the molar extinction of the product (ε mM pNA = 10.5) and c is the protein content in the sample (µg). The caspase activity was expressed in µmol pNA/min/mg of protein.

### 4.10. Statistical Analysis

The statistical processing was performed using computer programs Statistica 10.0 and GraphPad Prism 8.0.1. The data obtained in the study were tested for normality using the Shapiro–Wilk test. The nonparametric Mann–Whitney U-test and Kruskal–Wallis H-test were used to compare the obtained data; data were presented as median {upper quartile; lower quartile}. Differences were deemed statistically significant at *p* < 0.05, *p* < 0.01 and *p* < 0.001.

The research protocol was approved by the Local Ethics Committee of the Federal State Budgetary Scientific Institution Research Institute of Obstetrics, Gynecology, and Reproductology named after D.O. Ott (Protocol No. 107, 15 March 2021).

## 5. Conclusions

The data obtained in this study support the previously identified effects of MVs derived from NK cells in relation to changes in the functional and phenotypic characteristics of endothelial cells. Compared with unstimulated MVs, however, MVs derived from cells of the NK-92 cell line stimulated with IL-1β had a more pronounced inhibitory effect on endothelial cell migration, enhanced their activation and promoted their acquisition of a pro-inflammatory and procoagulant phenotype. MVs derived from cells of the NK-92 cell line stimulated with IL-1β, as well as MVs derived from unstimulated NK cells, are therefore able to influence the behaviour of cells and their response to external stimuli. However, the increased effects of MVs derived from stimulated NK cells compared with the effects of MVs derived from unstimulated cells suggests that MV functional activity depends on the microenvironment of parent cells and is modulated in response to factors and inducers that cause changes in the properties of source cells, particularly during an inflammatory response.

## Data Availability

The data presented in this study are available on request from the corresponding author.

## Supplementary Materials

The supporting information can be downloaded at: https://www.mdpi.com/article/10.3390/ijms222413663/s1.

## Abbreviations

Ac-DEVD-pNA: Ac-Asp-Glu-Val-Asp-pNA; Ac-LEHD-pNA: Ac-Leu-Glu-His-Asp-pNA Caspase-9 Chromogenic Substrate; DTT: Dithiothreitol; EDTA: Ethylenediaminetetraacetic acid; EC: Endothelial cell; FBS: Foetal bovine serum; MV: Microvesicles; PMA: Phorbol-12-myristate 13-acetate.
