# Supplementary figures and images for: Effects of Microvesicles Derived from NK Cells Stimulated with IL-1β on the Phenotype and Functional Activity of Endothelial Cells
Source: Int J Mol Sci. 2021 Dec 20;22(24):13663. doi: 10.3390/ijms222413663 (PMC8708902; doi:10.3390/ijms222413663)

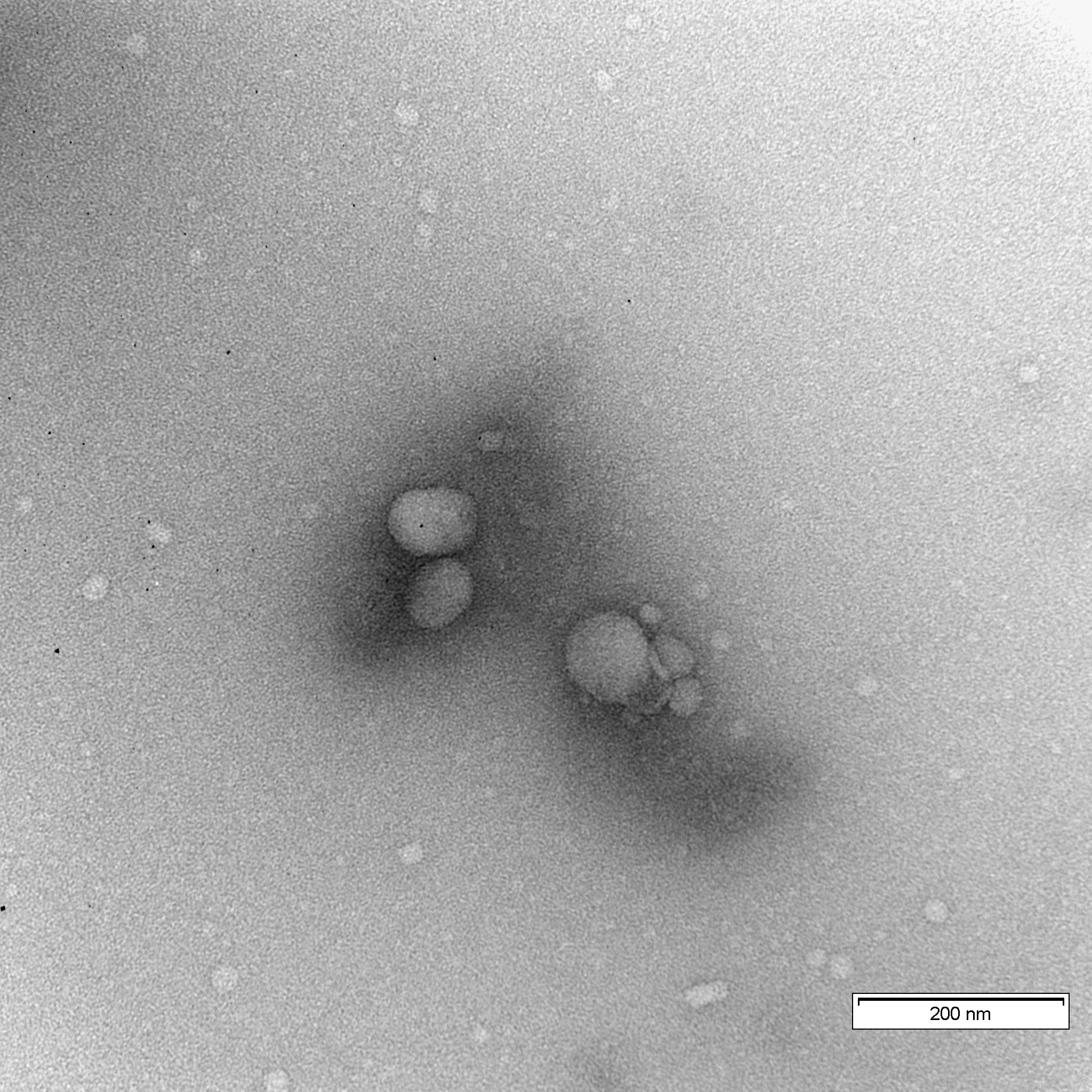

Supplement: Supplementary file 1 [file ijms-22-13663-s001.zip › Suppl Figure S1.TIF]

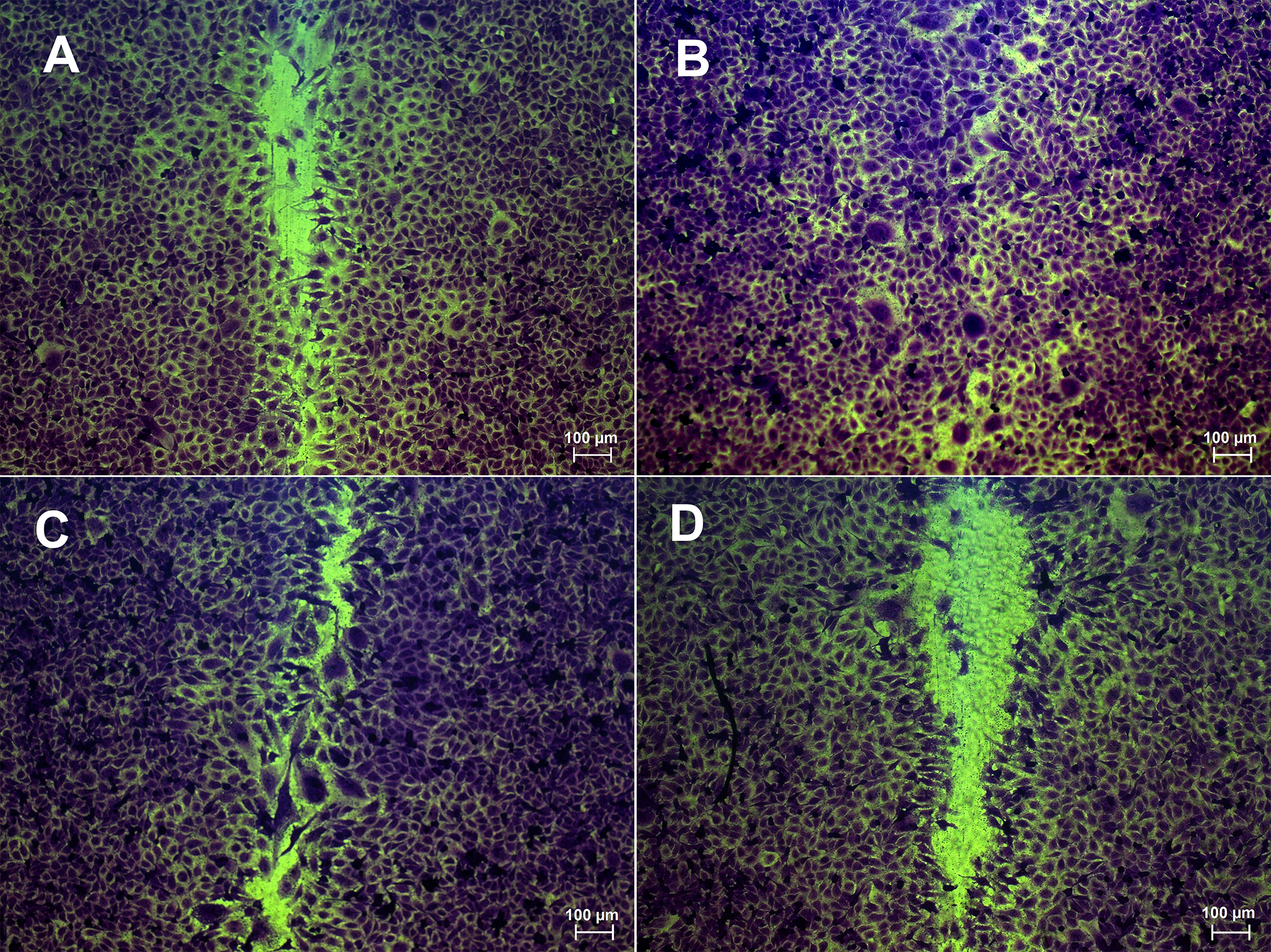

Supplement: Supplementary file 1 [file ijms-22-13663-s001.zip › Suppl Figure S2.tif]

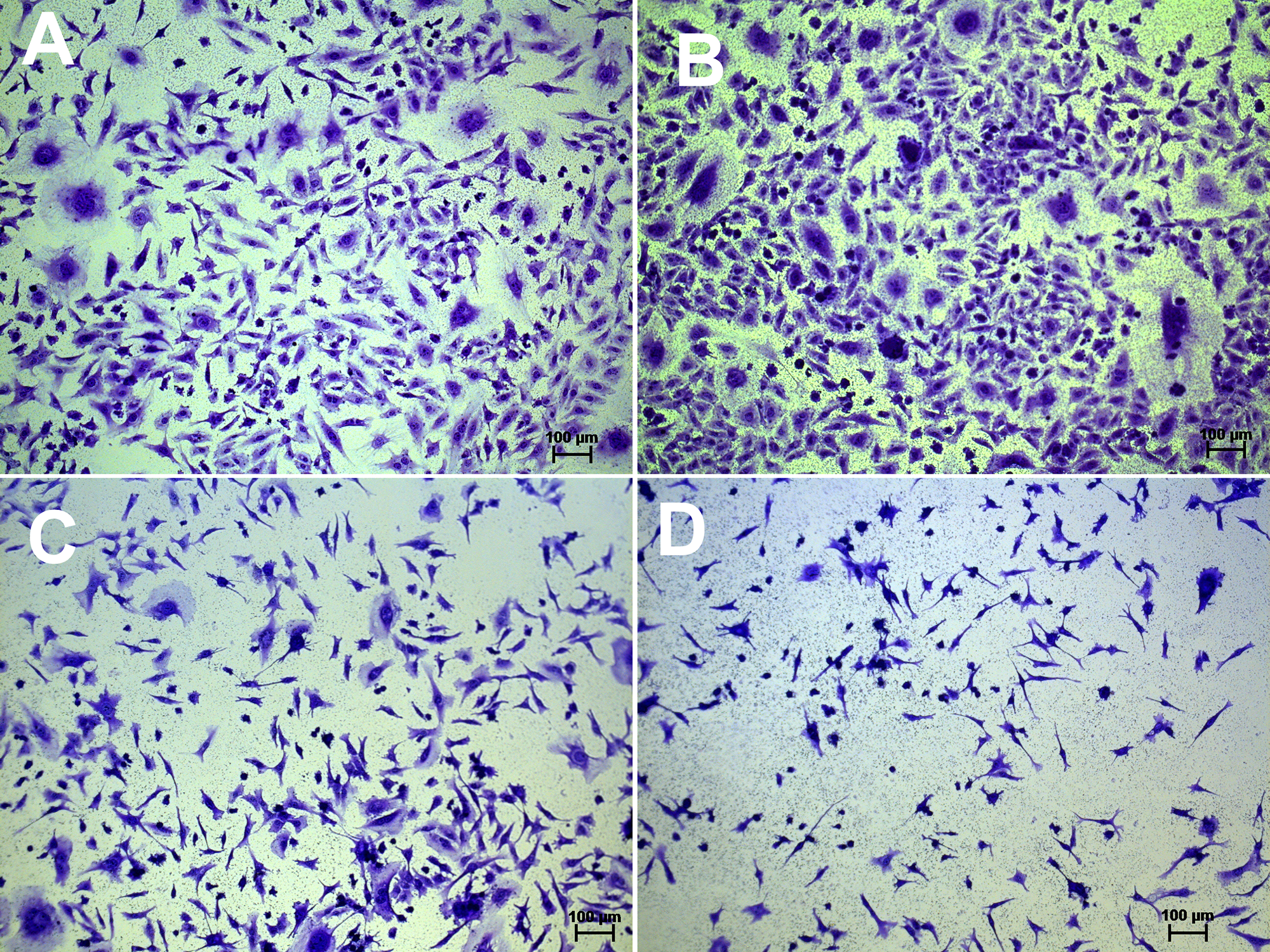

Supplement: Supplementary file 1 [file ijms-22-13663-s001.zip › Suppl Figure S3.tif]
